# Extracellular citrate and metabolic adaptations of cancer cells

**DOI:** 10.1007/s10555-021-10007-1

**Published:** 2021-12-21

**Authors:** E. Kenneth Parkinson, Jerzy Adamski, Grit Zahn, Andreas Gaumann, Fabian Flores-Borja, Christine Ziegler, Maria E. Mycielska

**Affiliations:** 1 Centre for Oral Immunobiology and Regenerative Medicine, Institute of Dentistry, Barts and the London School of Medicine and Dentistry, Queen Mary University of London, Turner Street, London, E1 2AD UK; 2 Institute of Experimental Genetics, Helmholtz Zentrum München, German Research Center for Environmental Health, Neuherberg, Germany; 3 Department of Experimental Genetics, Technical University of Munich, Munich, Germany; 4 Department of Biochemistry, Yong Loo Lin School of Medicine, National University of Singapore, Singapore, Singapore; 5 Eternygen GmbH, Berlin, Germany; 6 Institute of Pathology Kaufbeuren-Ravensburg, 87600 Kaufbeuren, Germany; 7 Department of Structural Biology, Institute of Biophysics and Physical Biochemistry, University of Regensburg, Universitätsstrasse 31, 93053 Regensburg, Germany

**Keywords:** Citrate, Warburg effect, OXPHOS, Redox, Senescence, Cancer-associated cells

## Abstract

It is well established that cancer cells acquire energy via the Warburg effect and oxidative phosphorylation. Citrate is considered to play a crucial role in cancer metabolism by virtue of its production in the reverse Krebs cycle from glutamine. Here, we review the evidence that extracellular citrate is one of the key metabolites of the metabolic pathways present in cancer cells. We review the different mechanisms by which pathways involved in keeping redox balance respond to the need of intracellular citrate synthesis under different extracellular metabolic conditions. In this context, we further discuss the hypothesis that extracellular citrate plays a role in switching between oxidative phosphorylation and the Warburg effect while citrate uptake enhances metastatic activities and therapy resistance. We also present the possibility that organs rich in citrate such as the liver, brain and bones might form a perfect niche for the secondary tumour growth and improve survival of colonising cancer cells. Consistently, metabolic support provided by cancer-associated and senescent cells is also discussed. Finally, we highlight evidence on the role of citrate on immune cells and its potential to modulate the biological functions of pro- and anti-tumour immune cells in the tumour microenvironment. Collectively, we review intriguing evidence supporting the potential role of extracellular citrate in the regulation of the overall cancer metabolism and metastatic activity.

## Introduction

We have recently discovered that cancer cells need extracellular citrate to support their metabolism [1]. Extracellular citrate uptake occurs through a plasma membrane citrate transporter (pmCiC), which is a variant of the mitochondrial citrate carrier belonging to the SLC25 gene family (mCiC; [2]). Importantly, we have also shown that extracellular citrate is needed for the metastatic progression of cancer and that citrate is supplied to cancer cells by cancer-associated stroma cells (CACs; [3]). Moreover, we have observed that cancer cells deprived of extracellular citrate release pro-inflammatory cytokines. Consequently, a significant increase of immune infiltration of tumours was observed. Interactions of tumour cells with the tumour microenvironment (TME) have gained considerable interest recently, and benign cells surrounding tumours have been determined to play a significant role in the progression of the disease. This occurs through the exchange of cytokines and metabolites but also interactions with the immune cells.

Extracellular citrate uptake induces changes in cancer metabolism which are reflected on many physiological levels. Increase in extracellular citrate uptake translates into a decreased intracellular citrate synthesis and allows for an optimal use of metabolic pathways which gives cancer cells several advantages. Potentially, this could increase their adaptability to different environmental conditions, therapy resistance and metabolic protection during dissemination and tumour dormancy.

In the present review, we will discuss the potential role of extracellular citrate in metabolic adaptations of cancer. In particular, we will concentrate on two major questions (1) whether extracellular citrate could play a role in inducing the shift from reductive to oxidative functioning of cancer cells and (2) whether organs rich in citrate could form “perfect niches” for metastasis. In this context, we will discuss the role of citrate synthesising cells such as senescent and cancer-associated cells in cancer progression, and the effect of citrate on the function of tumour microenvironment-associated immune cells.

## Use of glycolysis and OXPHOS by cancer cells

There are two major pathways used by cells to acquire energy, mitochondrial oxidation (OXPHOS) and glycolysis. OXPHOS is not only a very efficient energy supplier but is also responsible for keeping redox balance, the hallmark of a properly functioning metabolism. Not surprisingly, OXPHOS is the predominant way of acquiring energy by healthy cells. One of the main differences between cancer cells and healthy cells is the requirement of cancer cells to produce their own fatty acids for which citrate is the primary substrate [4]. Except for hepatocytes, normal cells do not produce fatty acids but take them up from the extracellular space; therefore, they do not need excess citrate synthesis and can use their Krebs cycle in the forward direction. Cancer cells need to produce fatty acids not only to be able to divide and replenish their plasma membrane but also to control activities of their plasma membrane proteins such as receptors or transporters [5, 6]. Fulfilling the special need for lipids, through increased intracellular citrate synthesis likely requires reversion and truncation of the Krebs cycle. This change in mitochondrial activity and citrate release into the cytoplasm has to be regulated, but the underlying regulatory mechanism has not been determined. It can be deduced that to produce excess citrate, cancer cells deprive themselves of an efficient ATP supplying mechanism in exchange for aerobic glycolysis (the Warburg effect).

Warburg effect was described around 100 years ago and assigned as the major pathway of acquiring energy by cancer cells. Despite its low efficiency in ATP synthesis, aerobic glycolysis offers some survival advantages to cancer cells. Firstly, using Warburg effect, cancer cells are able to produce excess citrate [7, 8]. Secondly, they are resistant to hypoxic conditions, which they encounter due to the rapid growth and metastatic progression [9, 10]. Finally, they produce and release excess lactate, which acidifies the cancer environment and allows for a faster tumour growth [11]. However, the use of aerobic glycolysis also has downsides. It requires more glucose uptake, which makes the cells less resistant to starvation and increases ROS levels [12, 13]. Increased ROS is beneficial at the early stage of tumour development and helps in transforming the surrounding stroma but also decreases cancer resistance to anti-cancer therapies [12]. Moreover, use of the Warburg effect requires substantial metabolic support including excess α-ketoglutarate, necessary to feed the Krebs cycle [14] and increased activities of the pentose phosphate pathway (PPP), malate-aspartate shuttle (MAS) and malic enzyme (ME) to keep redox balance [15].

Cancer cells also use aerobic glycolysis when oxygen is available; therefore, hypoxia is not the main reason for Warburg effect. On the other hand, the need for increased citrate synthesis from glutamine through the process of reductive carboxylation and reverse Krebs cycle through IDH (isocitrate dehydrogenase) activity could explain the truncation of the Krebs cycle [16] and so disabling its use for energy synthesis. Therefore, two questions arise. Firstly, would the lack of extracellular citrate be the reason for switching to glycolysis and secondly, could extracellular citrate play a role in switching between glycolysis and OXPHOS?

Although aerobic glycolysis seems to be an inefficient way of acquiring energy, it clearly offers cancer cells some necessary survival advantages. There are some tumour types with mutated IDH1 or 2, therefore unable, or with reduced capability, to produce citrate in the process of the reverse Krebs cycle or in the cytoplasm. Indeed, these tumours are unable to use aerobic glycolysis and consistently, show a decreased expression of HIF1α and several other elements of glycolysis such as SLC2A1, PDK1, LDHA, and SLC16A3 [17]. In consequence, mutated IDH leads to significantly reduced metastatic potential. Mutated IDH1 or 2 are frequently observed in brain tumours. Seventy percent of the grade II and grade III astrocytic and oligodendroglial gliomas and a small percentage of glioblastomas have the enzymes mutated (reviewed by [18]). Interestingly, all patients with IDH mutations (including glioblastomas) have a better prognosis than those with the wild type IDH [19]. In line with these observations, HCT116 colon cancer cells with mutated IDH1 showed significantly reduced ability to grow in vivo [20]. It is therefore clear that the inability to use Warburg effect reduces tumour growth and aggressiveness.

Cancer cells which also use OXPHOS, e.g. cancer stem cells (CSC), are well-known for an increased metabolic plasticity and are predominantly responsible for therapeutic resistance, metastatic spread, and disease recurrence [21]. CSCs are often quiescent with the ability for self-renewal and proliferative potential. They also show a high capacity to switch quickly between glycolysis and OXPHOS [22]. Although some of the CSCs such as breast, ovarian cancers or glioblastoma [23–25] rely on glycolysis as the predominant metabolic pathway, it is now more widely accepted that most CSCs use OXPHOS as their preferred mechanism of energy acquisition. In fact, CSCs from different cancer types rely mainly on OXPHOS with lower glycolytic activity, less lactate release and higher ATP synthesis (reviewed by [21]).

Based on the reviewed literature, it can be concluded that for the metastatic progression of the disease, cancer cells must have the ability to use both aerobic glycolysis and OXPHOS. While the ability to use Warburg effect is mandatory for aggressive types of tumours, features such as increased metastatic potential, therapeutic resistance, disease recurrence, and potentially dormant, or circulating, cancer cells seem to be more associated with OXPHOS.

## Role of citrate in cancer metabolism

Citrate plays a crucial role in cancer metabolism and the way it can be intracellularly synthesised has attracted a lot of research attention. Citrate can be synthesised in many different ways such as in the forward Krebs cycle, which can also be supplied with α-ketoglutarate derived from glutamine. However, in order to be released into the cytoplasm, citrate needs to accumulate in mitochondria and the citrate to isocitrate ratio needs to increase. To achieve this, cells need special regulatory mechanisms as explained below. Although a type of mechanism allowing for intra-mitochondrial citrate accumulation has not been identified in cancer cells, it is known that a defective electron transport chain or Krebs cycle leads to an increased use of reductive carboxylation from glutamine [26], which could be one such mechanism. It is also possible that a lack of extracellular citrate could stimulate the reverse Krebs cycle, or another unknown mechanism, allowing for excess citrate synthesis and release from mitochondria. Normal cells use citrate as an intermediate of the Krebs cycle and except for prostate epithelial cells, osteoblasts and astrocytes, increased mitochondrial citrate accumulation and release into the cytoplasm has not been observed. In prostate epithelial cells and osteoblasts, mitochondrial aconitase (mACN) has been determined to be a rate limiting enzyme which allows for citrate accumulation. Activity of mACN is decreased through increased Zn^2+^ levels [27]. Logically, therefore, if intracellular citrate synthesis was the primary aim of cancer metabolism and extracellular uptake would not play a significant role, epithelial-derived prostate cancer cells should keep this regulation allowing for intracellular excess citrate synthesis. Surprisingly, this is not the case. Prostate cancer cells lose the ability to uptake Zn^2+^ through downregulation of Zn^2+^ transporter expression, restoring full activity of the Krebs cycle [28, 29]. Therefore, cancer cells need fully active mitochondria and they must have a different mechanism allowing for excess citrate synthesis and release into the cytoplasm which remains to be elucidated.

We have recently determined that extracellular citrate is supplied to cancer cells by the cancer environment and that for cancer-associated cells citrate synthesis, and release is one of their major tasks [3]. Our findings are consistent with the notion that the choice of Warburg effect versus OXPHOS depends mainly on the metabolic interactions with the associated stroma. Extracellular citrate availability to cancer cells enhances metastatic progression through increased EMT/MET transition [3]. Moreover, in the presence of extracellular citrate, there was a clear reduction in intracellular amino acid levels consistent with a catabolic switch of cancer metabolism and increased mitochondrial ATP synthesis [3]. This catabolic preference is associated with the acquisition of a more aggressive phenotype in which a switch to OXPHOS and decreased overall metabolic activity give the cells a survival advantage in the midst of changing environmental metabolic conditions and starvation [30]. It can be therefore deduced that switching to OXPHOS is primarily responsible for the induction of the disease progression. Extracellular citrate availability would allow for a flexible use of OXPHOS or glycolysis with decreased intracellular citrate synthesis resulting in increased metastatic potential and resistance to therapies as already observed with the CSCs.

## Citrate in redox balance

The choice of metabolic pathways employed by cancer cells to support the redox balance depends on their preference towards glycolysis or OXPHOS. One reason for this preference might be that the use of OXPHOS allows for a better control of ROS and therefore, increased resistance to therapies. However, the following questions remain: how are OXPHOS, ROS control and extracellular citrate uptake related?

If we consider that reverse Krebs cycle leads to increased citrate synthesis, this could create an additional problem for keeping redox balance. There will be an increased need of NADPH for fatty synthesis and ROS control as well as increased need of NAD^+^ to sustain high rate of glycolysis. Lactate dehydrogenase (LDH), malate dehydrogenase (MDH) and MAS have been determined to be involved in controlling NAD^+^/NADH balance. Also, NADP^+^/NADPH balance was shown to be maintained by increased activities of the PPP oxidative branch and MEs.

### Malic enzymes

NADPH is essential to support fatty acid synthesis and to control ROS levels and therefore to increase cancer cell resistance to therapies. ME has been determined to play a particularly important role in this regard. There are 3 isoforms of the ME, two present in the mitochondria (ME2 and 3) and one in the cytoplasm (ME1). However, only ME1 and ME2 were considered to play a role in cancer metabolism. Mitochondrial ME2 using both NAD^+^ and NADP^+^ as cofactors is switched on by increased malate concentrations while increased fumarate levels further upregulate ME2 enzymatic activity [31]. ME2 overexpression has been associated with several cancer types including lung cancer, melanoma, or glioblastoma (reviewed by [32]). Interestingly, gastric cancer cells were shown to be more dependent on ME1 than ME2 in vivo under metabolic stress conditions [33]. Under these conditions, ME1 alone was able to account for the necessary NADPH and increase cancer cell survival. Moreover, silencing of ME1 resulted in strong dependency on glucose uptake [33]. Similar results confirming the role of ME1 under low glucose conditions were obtained on human cervical HeLa and large cell lung NCI–H460 cancer cells [34]. Accordingly, ME1 overexpression has been correlated with worse prognosis in hepatocellular, gastric and breast cancer patients [35–38] and increased lung cancer resistance to radiotherapy [39]. Moreover, ME2 depleted cells were more sensitive to glucose rather than glutamine deprivation with a strong dependence on pyruvate uptake suggesting more pronounced OXPHOS use [40]. Similar results were seen with other cancer types including melanoma [41] and glioma [42].

The fact that ME1 was found to be more important in supporting cancer survival in vivo and under low glucose conditions could be explained by the fact that ME1 being a cytosolic enzyme would be able to supply pyruvate to fuel OXPHOS without disturbing mitochondrial activity. On the other hand, ME1 could also supply pyruvate to LDH, which would increase NAD^+^ levels necessary to sustain glycolytic activity. Although, a truncated or reduced Krebs cycle under aerobic glycolysis conditions would be inefficient to sustain necessary malate levels for ME2, additional malate would be expected to enter mitochondria through the mCiC conducting citrate/malate exchange. Therefore, it is possible that the exact choice of the pathways depends on the particular needs of the cells and the way they are supported through their surroundings. For example, highly desmoplastic cancer types relying heavily on the interactions with the surrounding stroma, like gastric and pancreatic tumours, often have genomic deletions of ME2 [33, 43].

### Pentose phosphate pathway

The main pathway indicated in controlling the redox balance is the pentose phosphate pathway (PPP). PPP has been shown to have two branches, the oxidative branch, employed by cells which need increased NADPH supply, and the non-oxidative branch used by the cells which need to support proliferation through nucleotide precursors synthesis [44, 45]. Glucose-6-P dehydrogenase (G6PD) is the first enzyme of the oxidative branch. It exists as a dimer or monomer but is active only in its dimer form stabilised by NADP^+^ binding, while binding of p53 prevents dimerization of the enzyme [46]. The non-oxidative branch of the PPP generates ribose-5-P (R5P), necessary to sustain cell proliferation. If the cells need increased levels of NADPH, e.g. when using OXPHOS, R5P can be converted to glyceraldehyde-6-P followed by fructose-6-P, which then enters the gluconeogenesis pathway [45]. Interestingly, increased levels of cytosolic citrate taken up from the extracellular space would decrease phosphofructokinase-1 (PFK-1) activity down-stream of the G6PD. This could result in reduced activity of the non-oxidative PPP. Therefore, uptake of extracellular citrate might play a regulatory role in activating the oxidative PPP through (1) increased NADP^+^ levels resulting from increased fatty acid synthesis [1, 47], (2) as well as through inhibition of PFK-1. In this case, citrate taken up from the extracellular space could play a role not only in inducing OXPHOS and supporting fatty acid synthesis but also regulating metabolic pathways leading to a better protection of cancer cells against ROS. In line with this hypothesis, the rate of glycolysis was shown to be reduced and the oxidative PPP pathway upregulated by a TP53 inducible glycolysis and apoptosis regulator (TIGAR). Its expression increases O_2_ consumption and resistance to chemotherapies in cancer cells [48]. Simultaneously, a reverse process has been observed in cancer-associated fibroblasts (increased HIF, glucose uptake and LDH) consistent with increased citrate synthesis [3].

In line with the hypothesis that oxidative PPP activity is associated with the OXPHOS, some recent studies have shown that tumour types relying heavily on glycolysis (K-Ras-driven) have a relatively small percentage of glucose diverted to the PPP [49]. Moreover, silencing of the G6PD did not prevent formation of K-Ras-induced non-small cell lung, or HCT116 colorectal, primary tumour growth in mice and only slightly decreased triple-negative MDA-MB-231 breast cancer formation. However, G6PD silencing did moderately decrease lung colonisation when breast cancer cells were delivered through tail-vain injection [49]. A similar decrease of metastatic activity and no change of the primary tumour growth were observed when PPP activity was inhibited in melanoma [50], tongue cancer [51] or other oral squamous cell carcinomas [52].

K-Ras-driven tumours are known to rely heavily on glycolysis with increased glucose uptake, decreased Krebs cycle activity, increased glutamine use and reduced oxidative PPP [53, 54]. Therefore, increased use of the PPP would decrease glycolytic activity and so the lack of a substantial effect of G6PD silencing on the primary tumour growth. However, metastatic spread and colonisation were found to be more dependent on the oxidative PPP even in K-Ras-driven tumours [49]. The discussed data show clearly that oxidative PPP activity is associated with metastatic activity, while primary tumour growth is more oxidative PPP-independent. It could be therefore deduced that metastasising and colonising cancer cells require a switch to OXPHOS and decreased overall metabolism together with a stronger ROS control through increased NADPH levels. In this case, contribution of the oxidative PPP would be expected to play a more important role.

### Regulation of NAD + /NADH: lactate dehydrogenase, malate dehydrogenase, and the malate-aspartate shuttle

Lactate dehydrogenase (LDH) has been shown to play a major role in cancer metabolism. It metabolises the conversion of pyruvate into lactate accompanied by oxidation of NADH to NAD^+^. In this way, LDH supplies the NAD^+^ necessary to sustain glycolytic activity of cancer cells. Not surprisingly, silencing of LDH or use of LDH inhibitors inhibited tumour initiation and progression in vivo as well as increased oxidative stress, ROS synthesis and oxygen consumption [55]. Consequently, decreased LDH activity was shown to reduce cancer cell survival under hypoxic conditions [56]. However, some recent reports considered LDH activity alone as insufficient to support appropriate amounts of NAD^+^, due to the necessity of using glycolytic intermediates for biomass synthesis [57]. In this case, malate dehydrogenase (MDH) has been implicated in the maintenance of the appropriate NAD^+^ levels [58]. Blocking of MDH1 (cytosolic) resulted in decreased proliferation and glucose uptake in cancer cells. Consistently, overexpression of MDH1 is associated with a worse prognosis in several tumour types [58]. MDH1 and MDH2 (mitochondrial) are a part of the malate-aspartate shuttle (MAS) and convert oxaloacetate to malate with the concomitant oxidation of NADH [59]. The oxoglutarate carrier GC carries out exchange of malate against α-ketoglutarate, one of the elements of the MAS. Although the MAS has been shown to be coupled to the Krebs cycle, and likely to be downregulated under aerobic glycolysis, some studies show that silencing of the GC in KRAS^LA2^ lung tumour and melanoma decreased ATP synthesis and inhibited proliferation of the cells in vitro in addition to lung tumour formation in vivo [60]. Importantly, silencing of another element of the MAS, aspartate glutamate carrier 1 (SLC25A12), significantly reduced tumour growth by disrupting the NAD^+^/NADH ratio and aspartate levels [61]. Unexpectedly however, reduced activity of the MAS increased the metastatic activity of mouse Lewis lung carcinoma in vivo [61]. Consistently, AOAA, a specific inhibitor of the MAS, was shown to decrease ATP levels and proliferation rate through decreased glycolysis; however, it did not affect mitochondrial activity in C6 glioblastoma cells [62]. Although aerobic glycolysis decreases Krebs cycle activity, necessary for MAS/ME2 function [63], when increased levels of citrate need to be synthesised and released from mitochondria, malate levels could be restored by increased citrate/malate exchange through mCiC [64]. Another source of NADPH/NAD^+^ levels under increased mitochondrial citrate synthesis could be citrate itself. Citrate released into the cytoplasm could be cleaved by ATP-citrate lyase, followed by conversion of oxaloacetate into malate by MDH1 regenerating NAD^+^. Malate can then be transformed into pyruvate by ME1 restoring NADPH. Moreover, additional NAD^+^ for glycolysis could be generated by the conversion of pyruvate into lactate by LDH.

### Involvement of citrate in controlling redox balance

**Fig. 1:**
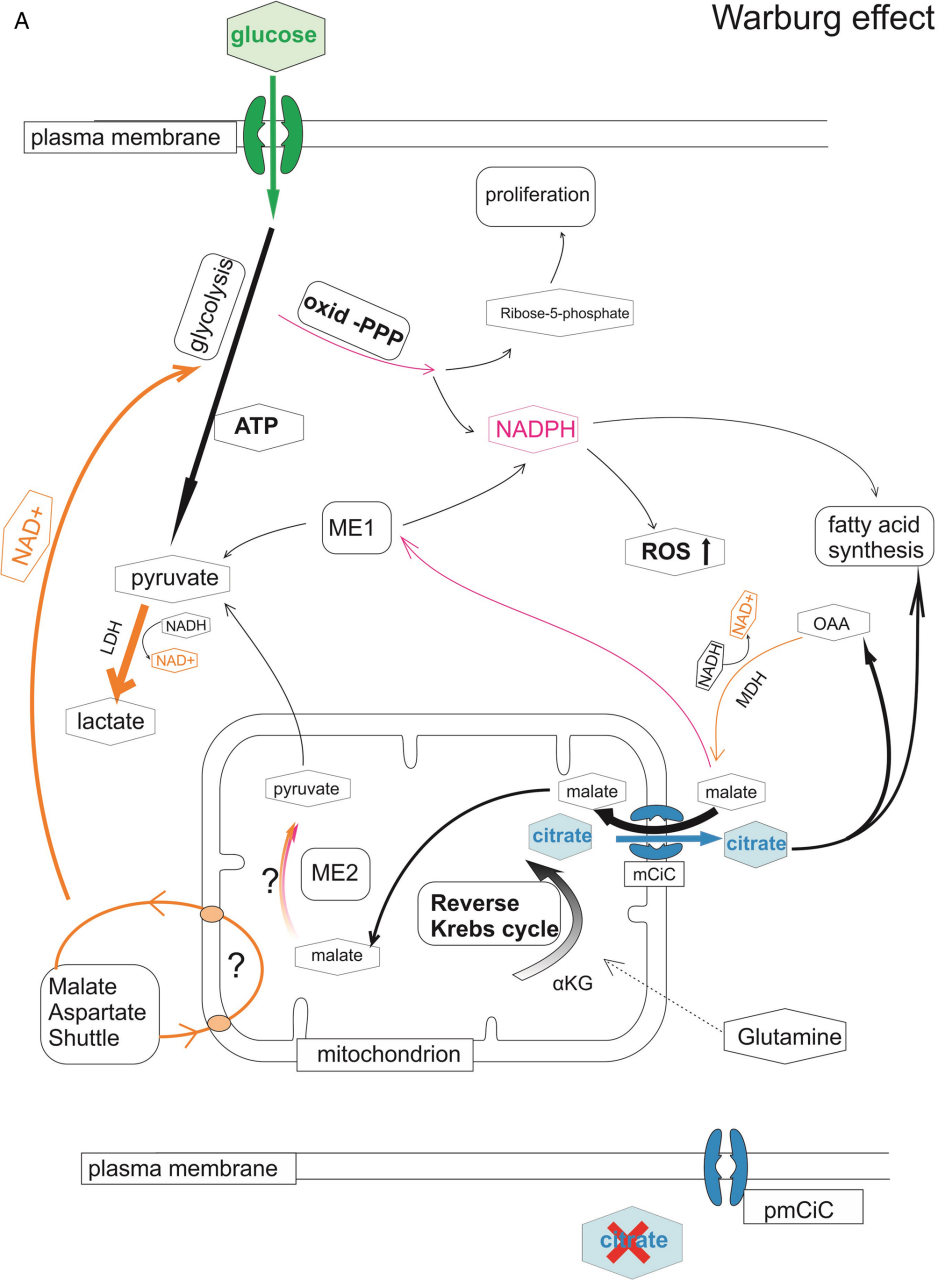

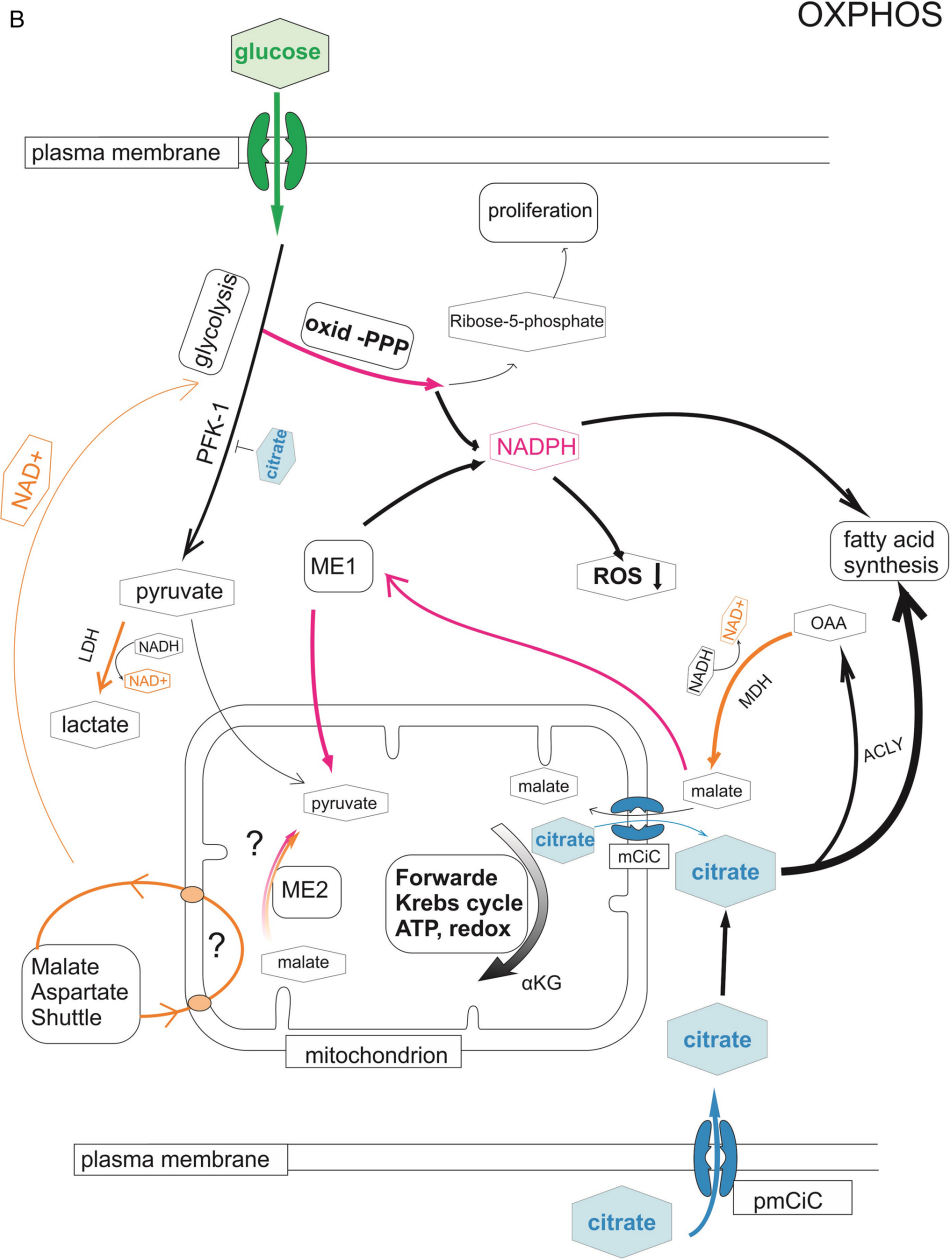
Involvement of citrate in Warburg effect versus OXPHOS. Diagram summarising the hypothesis that cancer cells potentially use different metabolic pathways in the presence or absence of extracellular citrate supported by the data discussed in the present review. (A) In the absence of extracellular citrate cancer cells need to synthesise excess citrate intracellularly in the process of the reverse Krebs cycle. In order to keep redox balance under these conditions, several different pathways to account for the needed NAD^+^/NADH and NADP^+^/NADPH are used. NAD^+^ necessary to support the process of glycolysis will be supplied mainly by LDH converting pyruvate into lactate. Because a part of the glycolysis intermediates is used to increase biomass, the action of LDH alone has been determined not to be sufficient. To increase NAD^+^ levels, citrate coming from mitochondria into the cytoplasm can be converted into oxaloacetate, a by-product of ATP-citrate lyase. MDH1 can further metabolise oxaloacetate into malate which can be then transported into mitochondria in exchange to citrate and support activity of the MDH2 (part of MAS) and ME2 contributing to the NAD^+^ pool. Alternatively, malate coming from the reaction of MDH1can also be further metabolised by ME1 to support NADPH level. Pyruvate, the product of ME1 can be further metabolised by LDH and supplying NAD^+^ for glycolysis. Under conditions without extracellular citrate oxidative PPP activity is likely to be decreased as it competes for G6P with glycolysis. Decreased PPP activity would lead to decreased NADPH level resulting in increased ROS. (B) On the other hand, when extracellular citrate is available, its uptake can support fatty acid synthesis in the cytoplasm allowing for the forward Krebs cycle and ATP synthesis in mitochondria. Decreased glycolytic activity will result in decreased need of NAD^+^ which might reduce the need for MAS activity allowing for undisturbed Krebs cycle. Decreased use of glycolysis will allow for increased NADPH supply through PPP leading to decreased ROS levels. Use of oxidative PPP will be also supported by increased levels of cytosolic citrate inhibiting phosphofructokinase (PFK-1). Increased cytosolic levels will allow for increased fatty acid synthesis. Oxaloacetate produced through the action of ATP citrate lyase can be further converted to malate, increase NADPH levels through ME1 and further supply pyruvate to mitochondria. Pathways contributing to NAD^+^ synthesis are depicted in orange, to NADPH in dark pink. Thickness of the lines represents activity level of the particular metabolic pathways. α-KG, α-ketoglutarate; ACLY, ATP citrate lyase; LDH, lactate dehydrogenase; ME1, 2, malic enzyme; MDH, malate dehydrogenase; MAS, malate aspartate shuttle, oxid-PPP, PPP oxidative branch; PFK-1, phosphofructokinase-1

Extracellular citrate uptake could therefore be a regulatory element supporting a switch of cancer metabolism to OXPHOS, as well as playing a role in increasing the protection of cancer cells against ROS (Fig. 1). In the absence of extracellular citrate, cancer cells are likely to use the reverse Krebs cycle to accumulate citrate in the mitochondria followed by its release into the cytoplasm. Malate coming into the mitochondria in exchange for citrate could sustain some activity of the MAS/ME2 enzymes providing an additional source of NAD^+^. Citrate coming into the cytoplasm could on the other hand be used to further provide NADPH through the action of ACLY/ME1 activity, followed by LDH conversion of pyruvate into lactate. On the other hand, extracellular citrate coming to the cytoplasm would allow for the forward Krebs cycle. Increased levels of citrate in the cytoplasm would upregulate the oxidative PPP pathway through increased levels of NADP^+^ derived from increased fatty acid synthesis as well as the blocking of PFK-1 activity. Cleavage of citrate by ACLY, the first step in fatty acid synthesis, could lead to NADPH and NAD^+^ synthesis through MDH/ME1 in a similar fashion to when extracellular citrate is absent. However, as extracellular citrate has been shown to increase fatty acid synthesis, this pathway could be expected to be more pronounced.

## Citrate — crucial metabolite in cancer development and metastatic progression?

The data discussed above suggest that metabolic preferences depend on cancer activities. The choice of the metabolic pathway might relate to the extracellular metabolic conditions. Therefore, potentially, metastasising cancer cells could show some preference towards organs with increased citrate level such as the liver, brain, bones and lung, which we will discuss in this section. Citrate plays a significant role in the metabolism of all cells. Besides its role in energy synthesis, it is also a chelator of the divalent cations including Ca^2+^ or Mg^2+^, and as such has an important function in several physiological processes such as neuronal excitability, blood clotting or kidney stone formation. The level of citrate in blood remains stable in a well-defined range between 100 and 150 µM [65] regardless of its intake with nutrition or fluxes from other tissues in particular from metabolically active soft tissues such as muscle or skin, after meals or exercise [66]. Bones are the major reservoir of citrate and it can be released into the circulation in the process of bone resorption. Excess citrate is removed by the kidneys and can be further absorbed and stored by bones [67].

Citrate is an important element of urine with a function of preventing bladder stone formation through Ca^2+^ and Mg^2+^ chelation. Using two different transporters NaDC1 and NaDC3 (SLC13 gene family), kidneys filter plasma citrate in the glomerulus tubules (NaDC3) and most of the citrate content is later reabsorbed in the proximal tubule (NaDC1; [68]).

Digested citrate is absorbed in small intestines and its level in blood increases after 30 min [69]. However, the absorbed citrate quickly accumulates in urine [70], consistent with the notion that keeping a stable concentration of citrate in blood is regulated through different mechanisms and does not depend on the food uptake. It therefore confirms the important role of citrate for a normal physiology.

**Fig. 2:**
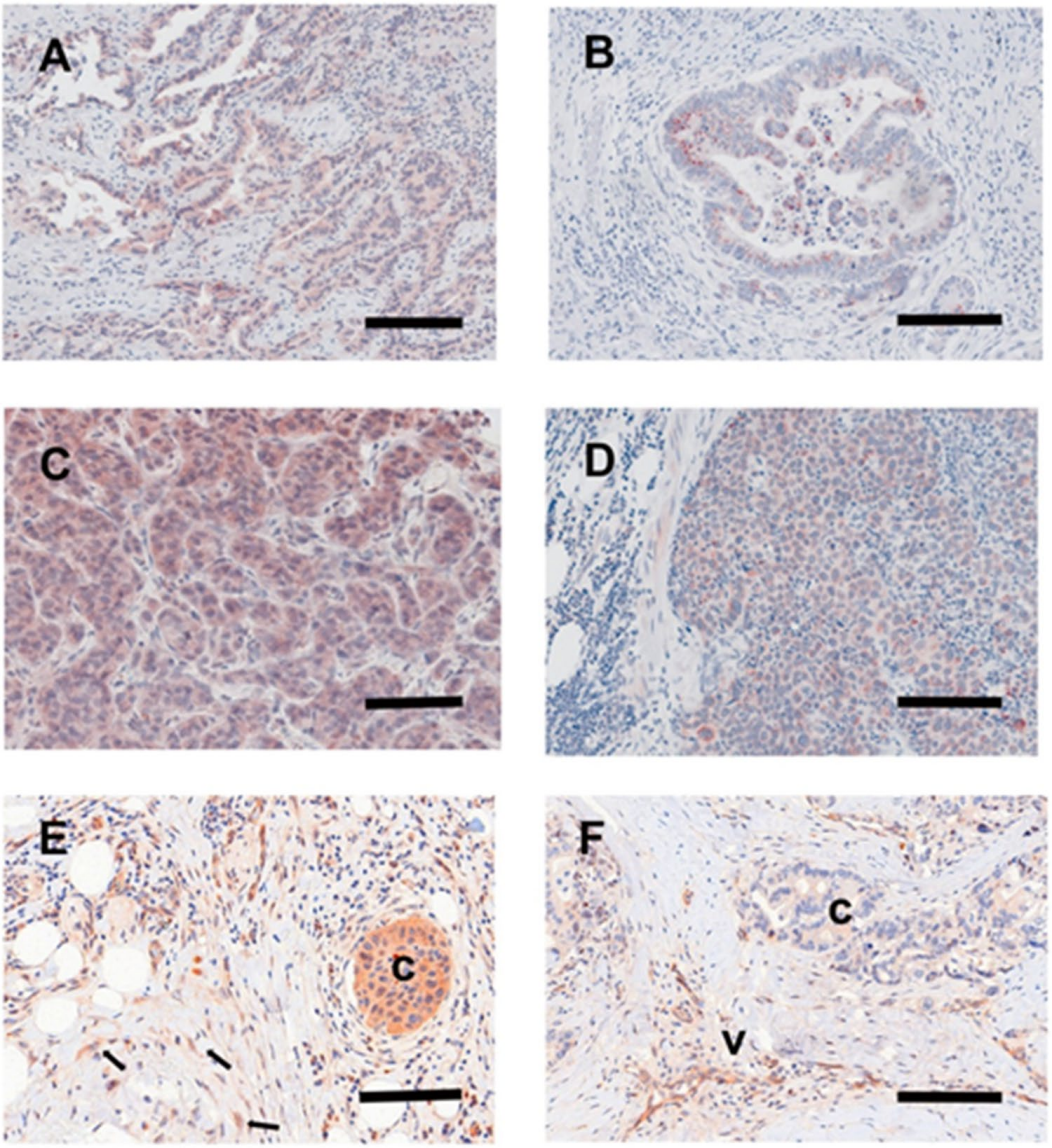
pmCiC is expressed in cancer cells and cancer-associated stroma in human tissues. Immunohistochemical expression of pmCIC investigated in a primary non-small cell lung carcinoma (NSCLC; A) showing moderate cytoplasmic signal in the carcinoma glands (DAB, magnification 100x; **—** 100 µm). B shows a colon carcinoma gland metastatic to the lung with a scattered expression of pmCIC (DAB, magnification 100x; **—** 50 µm). Strong uniform expression of a metastatic breast cancer (C) in a liver biopsy (DAB; magnification 200x **—** 50 µm). Weak expression of pmCIC in a metastasis (D) of a carcinoma in a lymph node (DAB, magnification 100x; **—** 100 µm). Figure E shows a solid pancreatic carcinoma (c) with surrounding desmoplastic stroma displaying a moderate (➔) as well as small vessels (DAB, magnification 100x; **—** 100 µm). Figure F shows a pancreatic cancer (c) complex with a prominent expression of pmCIC in small vessels (v) (DAB, magnification 100x; **—** 100 µm)

Interestingly, organs rich in citrate such as the bones, brain and liver are common sites for metastases. At the same time, tumours arising in these particular organs show minimal or no metastatic activities. Importantly, tumours arising in other organs like pancreatic cancer, urothelial carcinoma [71] or gastric cholangiocarcinoma [72] are known to be highly desmoplastic, therefore, more dependent of the associated stroma support (Fig. 2).

*Liver* is an organ known to attract metastasising cancer cells. It is responsible for citrate clearance from the plasma [73]. Similar to cancer cells, hepatocytes need citrate to produce fatty acids [74]. To take up extracellular citrate, hepatocytes express a citrate specific plasma membrane transporter NaCT, belonging to the SLC13 gene family [75]. Therefore, metabolically active hepatocytes show some similarities to cancer cells. It can be deduced that extracellular metabolic conditions in the liver might form a perfect niche for cancer/metastasis development.

*Brain* has significantly higher level of citrate compared to plasma reaching 400 µM. Citrate plays several functions in brain including being an energy product for neurons and regulating neuronal excitability through ion chelation. Astrocytes have been shown to synthesise and release citrate [76]; however, the exact mechanism of citrate synthesis and the origin of the protein responsible for citrate release have not been determined (reviewed by [77]).

*Bones.* With ~ 20–80 μmol/g, bones contain around 80% of body citrate. Therefore, the citrate concentration in bones is around 50 × higher than in soft tissues except for prostate and brain [78]. Citrate regulates the process of bone mineralisation [79]. The high content of citrate in bone has been associated with citrate synthetizing osteoblasts, which use a similar mechanism of m-aconitase inhibition [80] as secretory prostate epithelial cells. Bone stiffness is maintained by carbonated apatite imbedded in collagen [81]. These structures are studded with strongly bound citrate, necessary to keep stable thickness of the nanocrystals [81].

Bones are often hosts to metastases from tumours of different origin including the breast, prostate, lung or melanoma. Metastasising cancer cells produce osteoblastic and osteolytic lesions by disturbing the balance between osteoblast-mediated bone formation and osteoclast-mediated bone resorption [82]. Both types of lesions lead to several changes in bones including hypercalcemia [83, 84]. Ca^2+^ is chelated by citrate, and so increased Ca^2+^ levels in the presence of metastatic cancer cells would be consistent with the decreased level of citrate caused by consumption of this metabolite by cancer cells. Moreover, cancer cells can escape chemotherapy in bones by acquiring a dormant status [85] associated with the low metabolic activity seen in the presence of extracellular citrate [3]. Moreover, the interactions between cancer cells and cancer-associated bone cells play the major role in supporting metastasis formation [85] and osteoclastic activity might give tumour cells access to an inexhaustible source of citrate.

Although, extracellular citrate appears to be an important element for metastasising cancer cells, there are organs with increased citrate content like prostate gland which is a very rare place of the secondary tumour growth. Besides citrate, additional elements might also contribute to the formation of “perfect niches” for metastasising cancer cells, especially differences in immune cells activities. Bones are highly vascularised organs and enriched in suppressive or tolerogenic immune cells, such as regulatory T cells, M2 or tumour-associated macrophages [86], and myeloid-derived suppressor cells [87]. Contributing to the immunosuppressive environment, osteoclasts produce TGF-β that can inhibit T cell mediated anti-tumour responses (reviewed in [88]).

In regard to the brain, the central nervous system is rich in microglia and macrophages that maintain homeostatic conditions and control harmful responses mediated by cytotoxic CD8 + T cells [89]. In tumour development, upon establishment of metastatic cells, infiltrating myeloid-suppressor cells, granulocytes and monocytes outnumber T cells thus shifting conditions to generate an immunosuppressive environment [90]. The liver, on the other hand, filters blood and due to its primary task of fatty acid synthesis is particularly rich in several metabolites (including fatty acids) which can be of additional advantage to cancer cells. Interestingly, the lung, which is also a target of metastasising cancer cells, seems to have higher citrate levels than plasma [91]. Therefore, the prostate seems to be the only organ with increased citrate levels which is an uncommon place for the secondary tumour growth. Excessively high levels of citrate, which can be detrimental to cancer cells, could restrain metastasis at this site (please see the last section of the present manuscript). Moreover, the anatomical location of the gland and increased levels of hormones might also contribute to the absence of metastases. However, increased citrate levels, which could be one of the attractants for metastasising cells, should be studied further.

## Cancer-associated fibroblasts produce citrate to support tumour growth and metastasis

We and others have recently reviewed the evidence that the cancer environment can modulate the behaviour of tumour cells [92]. Here, we review the evidence of extracellular citrate being one of the key metabolites of the metabolic pathways present in cancer cells [93] and specifically there is now evidence that cancer-associated fibroblasts (CAFs) can be induced by prostate cancer cells when they are deprived of citrate and in turn the CAFs then supply the tumour cells with citrate to modulate the cytokines they produce [3]. The availability of citrate can induce epithelial-mesenchymal transition (EMT) in the short term to promote tumour cell invasion but longer-term exposure can do the reverse to promote the mesenchyme-epithelial transition (MET; [3]). The net result of this is to alter tumour cell metabolism [1] and to promote tumour growth, angiogenesis and metastasis [1, 3, 93].

Cancer cells have been shown to induce senescence and conversion to myofibroblasts in neighbouring fibroblasts and senescent cells are present in naturally occurring CAFs. This appears to occur via ROS and TGF-β, and in the case of squamous cell carcinoma, this appears to be specific to tumour cells that have by-passed senescence (see below) and become genetically unstable [94].

## Senescent cells in the cancer environment as potential suppliers of extracellular citrate

**Fig. 3:**
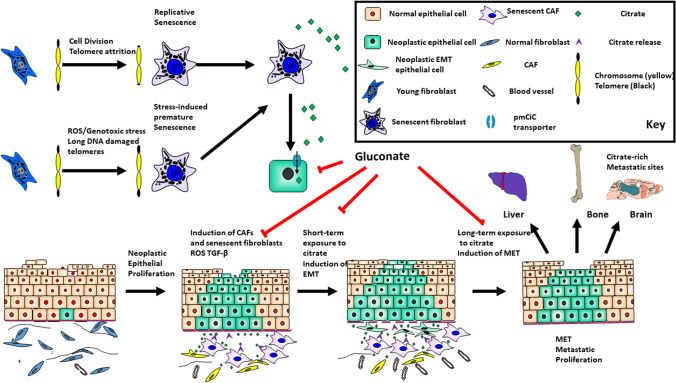
Citrate in tumour invasion and metastasis: the role of CAFs and senescence. The cartoon summaries the role of fibroblast activation and senescence in the modulation of carcinoma behaviour during tumour progression. Senescence can occur following proliferative exhaustion or more acutely following cellular stress and the latter also induces fibroblast activation and the formation of CAFs which secrete (purple arrowheads) extracellular citrate (green diamonds). Developing cancer cells induce both of these phenotypes by secreting ROS and TGF-β and in turn CAFs (yellow) and senescent fibroblasts (purple) deliver citrate and other factors to induce EMT (green spindle-shaped cells) and angiogenesis to promote invasion into the adjacent mesenchyme. This is achieved in part by the upregulation of pmCiC (blue) on the developing cancer cell surface (green shaded cells with black nuclei). Many favoured metastatic sites are citrate-rich and long-term exposure to citrate enhances MET and may aid the growth of metastatic deposits at these sites (arrows). The pmCiC inhibitor gluconate (red lines) mutes both tumour proliferation [1, 141] and metastatic spread [3] to support this hypothesis

As discussed above, organs rich in citrate might be attractive niches for secondary tumour growth but cancer cells can also import citrate from their surroundings and one potential source is senescent cells that are commonly found in the cancer environment as part of the CAF population [94, 95] especially following cancer therapy ([96]; summarised in Fig. 3).

Classical cellular senescence was originally defined as an irreversible cell cycle arrest that was distinct from quiescence, terminal differentiation and apoptosis and can occur following multiple rounds of cell division (replicative senescence [97] or following a wide range of cellular stresses such as ionising radiation, signalling imbalance and oxidative damage otherwise known as stress-induced premature senescence (SIPS) [98, 99]. In addition, a phenotype resembling cellular senescence occurs in post-mitotic tissues such as the liver and brain following cellular stress and during chronological ageing [100–102].

Interestingly, the classical senescent fibroblast phenotype resembles that of cancer cells in that energy metabolism is shifted away from OXPHOS towards glycolysis and the PPP in the presence of pyruvate [103]. Furthermore, many of the other metabolic enzymes and pathways discussed above including MAS (ME2) and IDH1/2 also regulate the senescent phenotype and the role of these pathways has been extensively reviewed recently [104].

For example, ME1 and ME2 expression declines in senescent fibroblasts and overexpression of either counters senescence. The above phenotypic effects are possibly mediated by the positive effects of ME1/2 on NADPH and so their loss in senescence could reduce NADPH-dependent functions, especially antioxidant defences in the case of ME2 [105]. Furthermore, there is a reciprocal relationship between ME1/2 and p53 [103] which can regulate both glycolysis and the PPP [104, 106].

MDH1 and other components of the MAS also decline in senescence and lower the NAD^+^/NADH ratio. Similarly, the inhibition of both IDH1 [107] and IDH2 [108] promotes senescence through the upregulation of ROS to cause SIPS-like senescence. Notably, these phenotypes are rescued by pyruvate, as is mitochondrial-dysfunction-induced senescence (MiDAS) regulated by sirtuins 3 and 5 [104]. Of importance here, extracellular pyruvate is important for the optimum release of extracellular citrate (EC) from senescent fibroblasts (James and Parkinson –unpublished data). Moreover, pyruvate from CAFs can promote the survival of certain cancer cells by upregulating the Krebs cycle [109] and also promote metastasis by inducing the production of α-ketoglutarate and remodelling the extracellular matrix [110] but the role of pyruvate in the suppression of mitochondrial function in these models was not examined.

## Citrate export and its upregulation in senescent fibroblasts

When fibroblasts undergo classical senescence, EC accumulates up to 11-fold. In the same cells, citrate becomes depleted in parallel with a shift in metabolism away from the Krebs cycle and towards glycolysis, consistent with high levels of citrate being incompatible with the latter as citrate antagonizes glycolysis by inhibiting PFK [91]. However, it is still not clear whether citrate export increases or import declines.

When cancer cells and fibroblasts are in close contact lactate is released by CAFs and senescent cells and transferred to the cancer cells via the monocarboxylate transporter SLC16A3/MCT4 to drive oxidative phosphorylation and tumour progression via what has been termed ‘the reverse Warburg effect’ [111, 112]. In this instance, senescence is of the SIPS type because the target cells were skin fibroblasts immortalised by telomerase and therefore would not exhibit replicative senescence but senescent fibroblasts induced by ionising radiation (SIPS) also produce more extracellular citrate [103].

## Citrate transport in epithelial tissues and its uptake by a wide range of cancer types

Citrate is transported into the cytoplasm from the mitochondrion via SLC25A1 (CiC) in exchange for malate [93] and is then exported out of the cells in prostate epithelia by a variant of SLC25A1 (pmCiC) located at the plasma membrane instead of the mitochondrial membrane. mCiC and pmCiC have alternative first exons and differ only in the first 38 amino acids of the N-terminal domain [2]. Another plasma membrane transporter encoded by the *ANKH* gene (SLC62A1) is also expressed in the prostate and has recently been shown to export citrate, in addition to malate, succinate and phosphate [113]. However, our knowledge of plasma membrane transporters and their role in the transport of the Krebs cycle metabolites is far from complete.

In mitochondria, mCiC exchanges citrate against the Krebs cycle intermediate malate. This 1:1 electroneutral antiport is driven by the malate gradient between cytoplasm and mitochondria. The transporter domain of pmCiC is 100% identical to mCiC. If pmCiC also functions as a citrate/malate exchanger, the question remains whether pmCiC would facilitate an import or an export of citrate. Secondary transporters theoretically can switch between import and export as substrates are able to bind from either side of the membrane to the central binding site although with altered affinities. In line with the questionable functional purpose of having a citrate antiporter in the plasma membrane, pmCiC is generally not expressed at all or only in very low amounts in the plasma membrane of normal cells. Most interestingly, the functional impact of pmCiC changes dramatically in cancer cells. In fact, expression is upregulated in a wide variety of cancer tissues [1] (see also Fig. 2) with a clear directionality of citrate transport into the cell where pmCiC imports citrate from the outside into the cytoplasm following a citrate gradient. As pmCiC working as antiport would not be physiologically unreasonable, there might be the possibility that pmCiC switches from antiport to a citrate uniport. A transport mode switch is not spontaneous but can be triggered by mutations or changes in interaction with the membrane and/or proteins [114]. Here, the N-terminal domain of pmCiC comprising several positively charged residues is one of the prime suspects for such functional interaction switch. From a mechanistic point of view, a citrate uniport would require that pmCiC cycles back to an outward facing open state spontaneously, which would only happen if this state is energetically favoured. The N-terminal domain could interact directly with key residues that drive the conformational changes from inside to outside changing the conformational energetic landscape in a way that the pmCiC returns fast to the outward facing state. The accessibility of the pmCiC central binding site would allow for such an interaction. However, it is also possible that an interaction of the N-terminal domain with the surrounding membrane could switch the transport mode. Further structure–function studies are required to shed light into the mechanistic differences between pmCiC and mCiC.

## The role of citrate on tumour-infiltrating immune cells

Tumour-infiltrating innate and adaptive immune cells are a prominent feature of the tumour microenvironment (TME), and consist of different subpopulations, including cytotoxic CD8 + and helper CD4 + T cells, regulatory T and B cells (Tregs and Bregs), natural killer (NK) cells, macrophages, and myeloid-derived suppressor cells (MDSCs; [115, 116]). Originally, it was believed that immune cells in the TME were associated with enhanced anti-tumour responses and better prognosis. However, it has been now shown that immune cells can display pro- or anti-tumour effector functions, with the final outcome being tumour suppression or progression and metastasis, depending on the number, spatiotemporal distribution of relevant immune cells, and the type and/or stage of cancer.

Intercellular communication between tumour and immune cells, either through direct cell–cell interactions or the release of soluble cytokines and growth factors, is the basis of mechanisms that tumour cells use to induce tolerance and suppression of anti-tumour responses [117, 118]. Adding to this complex scenario, recent studies have highlighted the important role of the metabolic state within the TME [119]. Because of their high energy requirements, tumour cells can deprive nutrients and/or release metabolic products with the potential to either exhaust and induce non-responsiveness of anti-tumour effector cells or enhance and sustain the function of tolerance-inducing immune populations [120]. For instance, using a mouse model of sarcoma, Chang and colleagues found that the high energy requirements of tumour cells trigger a competition for glucose that results in blocking of glycolysis in IFNγ-producing effector T cells and tumour progression [121]. Glucose deprivation would favour then the development of regulatory Tregs which rely on OXPHOS and lipid oxidation metabolism [122]. Other metabolic products contributing to the establishment of an immunosuppressive TME include adenosine and lactate. Recent evidence has highlighted the role of these metabolites in the induction and activation of pro-tumour immune cells such as Tregs and M2-like macrophages, and dendritic cells with an abnormal accumulation of lipids [123–125].

As mentioned earlier, citrate is a critical metabolite that tumour cells require to sustain their metabolism. In addition, recent evidence points to the important role of citrate in immune cells. Once believed to be a mere metabolic by-product, citrate is now considered an important modulator of the activity of immune response [126]. Citrate metabolism might have an important effect on tumour-infiltrating immune cells. Once exported from mitochondria, citrate enters metabolic pathways important for fatty-acid synthesis, protein acetylation [127], and contribute to the mechanisms generating the energy required to fight tumours off [128]. It has been shown that NK cells express high levels of citrate and treatment with inhibitors of ATP citrate lyase, impaired the cytotoxic function of NK cells [129]. Citrate, and products derived from its metabolism, are also required for differentiation, and proliferation of effector IFNγ-producing T cells, secretion of TNFα, and nitric oxide, prostaglandins, and IL-1 induction by macrophages (reviewed in [130–132]). Observations that ACLY inhibition results in decreased production of antibodies indicate that citrate might play an important role in B cell-mediated anti-tumour response [133].

As shown in the above-mentioned studies, the metabolic reprogramming of immune cells depends on mitochondrial-derived intracellular citrate. However, the effect of extracellular citrate on immune cells at the TME is a topic that deserves further research. It might be possible that effector and suppressor immune cells uptake from, or contribute to, the extracellular pools of citrate in the TME. Work by our group and others has shown that tumour cells respond to variations in citrate concentration. This has led to the suggestion that manipulation of citrate levels in the TME might have important effects on both tumour and immune cells and result in therapeutic effects. In fact, recent studies with a mouse model of cancer have shown that tumour regression after therapeutic administration of citrate was associated with a cytokine- and T cell-mediated anti-tumour response [134]. These points need more in vitro and pre-clinical in vivo studies of the effects of artificial manipulation of TME citrate levels.

## Extracellular citrate a double-edged sword in cancer metabolism

We have presented evidence and put forward a hypothesis that extracellular citrate might play a major role in cancer metabolism and is responsible for a switch between Warburg effect and OXPHOS. There is evidence that reducing physiological levels of citrate uptake in some cancer cells inhibits proliferation in vivo and in vitro [1, 135] and metastasis and angiogenesis in vivo [3]. However, citrate levels can reach 50 mM in seminal fluid and up to 180 mM in prostatic fluid [136]. While these high citrate levels may only apply to prostate cancer, for the completeness of this review, it has to be also mentioned that extracellular citrate has been postulated to have detrimental effects on cancer cells if supplied at high concentrations. Indeed, severely increased intracellular levels of citrate are likely to disturb cancer cells metabolism through glycolysis inhibition [134, 137]. Specifically, when incubated with 10 mM citrate, cancer cells were shown to have decreased proliferation, ATP synthesis, increased apoptosis and sensitivity to cis-platin [137–139]. Moreover, oral application of high doses of citrate in vivo decreased tumour growth and increased immune infiltration [134]. Although, these approaches seem contradictory, they are based on the same ability of cancer cells to take up extracellular citrate. While physiological levels of extracellular citrate are highly beneficial for cancer cells, high concentrations of citrate cause detrimental effects [140]. Both approaches should be therefore considered when looking for novel anti-cancer therapies.

## Conclusions

Based on the data highlighted in this review, we propose a hypothesis in which cancer cell metabolic preference depends largely on the availability of extracellular citrate. We postulate the role of extracellular citrate as the switch between glycolysis and OXPHOS. Understanding the role of extracellular citrate and the mechanisms through which citrate is produced and supplied to cancer by CAS and the TME would potentially allow for manipulation and control of cancer metabolism.

## Data Availability

Not applicable.
